# Delayed Oxaliplatin-Related Severe Neurotoxicity in Metastatic Colorectal Cancer: A Case Report

**DOI:** 10.7759/cureus.33578

**Published:** 2023-01-10

**Authors:** Yohei Iimura, Naoki Furukawa, Seiichiro Kuroda

**Affiliations:** 1 Department of Pharmacy, IMSUT Hospital, Institute of Medical Science, University of Tokyo, Tokyo, JPN

**Keywords:** colorectal cancer, chemotherapy, oxaliplatin, coasting phenomenon, peripheral neuropathy

## Abstract

In patients receiving oxaliplatin-based chemotherapy, resulting in frequent peripheral neuropathy and requiring long-term management, anticancer drug-induced platinum-based peripheral neuropathy (mixed motor, sensory, and autonomic neuropathy) can result in the coasting phenomenon in which the symptoms worsen temporarily after two to three weeks, even after the cessation of the drug. The coasting phenomenon is difficult to manage due to the unpredictable nature of the symptoms. We encountered a patient with grade 3 peripheral neuropathy that developed rapidly in the second cycle after the treatment to switch from mFOLFOX6/bevacizumab to FOLFIRI/aflibercept. Supportive care with duloxetine was unsuccessful in this patient. Herein, we report the case.

## Introduction

Oxaliplatin is a key platinum anticancer agent used in chemotherapy for the treatment of colorectal cancer. Peripheral neuropathy is a typical adverse effect of oxaliplatin. Oxaliplatin-related peripheral neuropathy is classified as acute neuropathy, which develops within a few hours of administration, and cumulative neuropathy, which appears with repeated administration. The frequency of peripheral neuropathy is high; it is observed in more than 90% of patients receiving oxaliplatin. More than 30% of patients receiving oxaliplatin have grade 2 (impairment of motor or sensory function, but not affecting daily life) symptoms, and more than 10% have grade 3 (impairs motor or sensory function and affects daily life) symptoms [1] (evaluated by National Cancer Institute’s Common Toxicity Criteria, version 1 [2]). The frequency increases above total doses of 800 mg/m2 [3].

Additionally, according to the data from the MOSAIC trial (a clinical trial evaluating whether adding oxaliplatin to continuous infusion fluorouracil plus leucovorin as adjuvant chemotherapy prolongs disease-free survival of resectable colon cancer; the safety of the regimen was evaluated as a secondary endpoint) on adjuvant chemotherapy containing oxaliplatin, peripheral neuropathy persists in more than 15% of patients and has been shown to persist for up to four years after chemotherapy [1]. In the MOSAIC trial, more than 90% of the patients developed peripheral neuropathy during chemotherapy, while those with advanced recurrent colorectal cancer required long-term management. Oxaliplatin-related peripheral neuropathy requires a longer recovery period with a median recovery time of 13 weeks for grade 3 peripheral neuropathy [3]. Since peripheral neuropathy can persist for a long time, it is one of the causes of reduced quality of life. Platinum anticancer drug-induced peripheral neuropathy can result in the coasting phenomenon in which the symptoms worsen temporarily after two to three weeks, even after the cessation of drug therapy [4-5]. Since the onset of the symptoms cannot be predicted, using a "stop-and-go strategy" (a treatment strategy to stop the administration of oxaliplatin before peripheral neuropathy becomes severe) [6] is not effective against the coasting phenomenon. Reports on oxaliplatin-related coasting are limited. We report a case of rapid-onset severe peripheral neuropathy following oxaliplatin-based chemotherapy.

## Case presentation

A 74-year-old man diagnosed with stage IV rectal cancer with extraserosal infiltration (performance status: 0; fully active, able to carry on all pre-disease performance without restriction). He had no underlying disease and no concomitant medication, and his major organ functions were preserved. He was administered six courses of mFOLFOX6. Although the chemotherapy was temporarily postponed due to grade 2 peripheral neuropathy (mixed motor and sensory neuropathy) confirmed by the patient’s self-report (evaluated according to Common Terminology Criteria for Adverse Events version 5.0. [2]), peripheral neuropathy disappeared thereafter, and a total of 13 courses were administered over nine months without the patient experiencing any symptoms. However, after the regimen was changed from mFOLFOX6/bevacizumab (fluorouracil continuous infusion/levoleucovorin/oxaliplatin/bevacizumab) to FOLFIRI/aflibercept (fluorouracil continuous infusion/levoleucovorin/irinotecan/aflibercept), due to disease progression, numbness in the limbs suddenly occurred in the second cycle (five weeks after the discontinuation of oxaliplatin).

**Figure 1:**
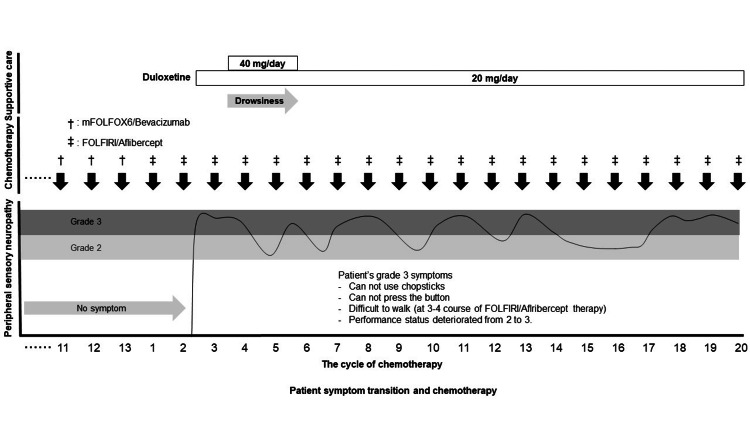
Patient transition and chemotherapy. Abbreviations: mFOLFOX6, oxaliplatin (85 mg/m2), levofolinate (200 mg/m2), and bolus 5-fluorouracil (400 mg/m2) on day 1 followed by 46-hour continuous infusion of 5-fluorouracil (2400 mg/m2); FOLFIRI, irinotecan (150 mg/m2), levofolinate (200 mg/m2), and bolus 5-fluorouracil (400 mg/m2) on day 1 followed by 46-hour continuous infusion of 5-fluorouracil (2400 mg/m2). Side effect grades were evaluated using the Common Terminology Criteria for Adverse Events (CTCAE) version 5.0. mFOLFOX6/bevacizumab and FOLFIRI/aflibercept regimens were administered once every two weeks without postponement.

The severity of the peripheral neuropathy experienced by the patient was identified as grade 3, five days after the commencement of chemotherapy. The patient’s grade 3 symptoms included (1) the inability to use chopsticks, (2) press buttons, or (3) walk (after three to four courses of FOLFIRI/aflibercept therapy); at the time of the most severe symptoms, the patient's performance status deteriorated from grade 2 (ambulatory and capable of all selfcare but unable to carry out any work activities for approximately more than 50% of waking hours) to grade 3 (capable of only limited selfcare and confined to bed or chair for more than 50% of waking hours). The numeric rating scale score range for pain (spontaneous numbness pain) in the limbs was 7-8. Duloxetine (40 mg/day) was administered as supportive care for peripheral sensory neuropathy. Subsequently, although the symptoms temporarily improved to grade 2, the patient developed drowsiness. After reducing the duloxetine to 20 mg/day, the symptoms worsened again from grade 2 to 3. This patient had been administered a total of 20 courses of FOLFIRI/aflibercept therapy, and the peripheral neuropathy, which remained at grade 2-3, showed no signs of improvement (Figure 1). Supportive care with duloxetine in this patient was not effective. He is still alive without any tumor exacerbation, however, his neuropathy did not improve, and the same degree of symptoms persisted. The patient refused to take medications due to their side effects, therefore dose up of duloxetine was not administered. Additional oral administration of other gabapentinoids was not administered due to the patient's refusal of supportive care medications.

## Discussion

In this case, the total dose of oxaliplatin administered was 1457 mg/m2. The patient had no history of diabetes or neurological disorders. At the onset of severe peripheral sensory neuropathy, the serum calcium level was 9.0 mg/dL (normal range: 8.8-10.1 mg/dL), serum magnesium level was 2.2 mg/dL (normal range: 1.8-2.4 mg/dL), and the serum ammonia level was 31 μg/dL (normal range: 30-80 μg/dL). Considering the serum vitamin levels, the vitamin B12 level was 329 pg/mL (normal range: 180-914 pg /mL) and the thiamine level was 47 ng/mL (normal range: 24-66 ng /mL). Standard peripheral neuropathy prevention strategies were explained to the patient.

The frequency of peripheral neuropathy increases when the total dose of oxaliplatin is above 800 mg/m2 [3]. This patient was a rare case with few late-onset symptoms that occurred when the total dose exceeded 800mg/m2.

The "stop-and-go" strategy [6] has been reported as a useful countermeasure against oxaliplatin-induced peripheral neuropathy and is used widely in clinical practice. The OPTIMOX 1 study compared the administration of six courses of FOLFOX therapy followed by 12 courses of oxaliplatin-free maintenance therapy and the subsequent reintroduction with the continuation of FOLFOX therapy, until disease progression. In this study, while the use of the stop-and-go strategy reduced the neurotoxicity to grade 3, there was no difference in the antitumor effect between the two groups [6]. However, this strategy was not useful in cases such as ours in which the peripheral neuropathy worsened rapidly after the cessation of oxaliplatin administration. Since there were no or only mild, self-manageable symptoms of peripheral neuropathy during the administration of oxaliplatin, there was no reason for the discontinuation of oxaliplatin. Although duloxetine [7] and pregabalin [8] have been demonstrated to be effective supportive agents, caution is recommended in the use of both drugs in view of their side effects such as drowsiness and nausea. In Japan, the maximum daily dose of duloxetine is 60mg; however, our patient could not be administered the maximum dose because of the side effect of drowsiness. In the summary of product characteristics [9], according to drug-drug interaction, bevacizumab is able to enhance sensory neuropathy symptoms in the presence of paclitaxel or oxaliplatin. Although to the best of our knowledge, there is no report on aflibercept [10] as described above, we surmise aflibercept may have the same effect since it has the same vascular endothelial growth factor receptor inhibitory effect. Although the exact mechanism is unclear, bevacizumab and aflibercept, which also cause vascular damage, may exacerbate oxaliplatin-induced peripheral neuropathy.

Cisplatin and oxaliplatin are more likely to cause the coasting phenomenon than other chemotherapy-related inducers of peripheral neuropathy [11]. However, the number of reports is limited, and standard supportive care for peripheral neuropathy and the coasting phenomenon have not been established. In this patient, the administration of duloxetine was ineffective against the coasting phenomenon. Further studies are necessary to determine the best care for patients who experience a platinum-related coasting phenomenon. A limitation of this study is that electrodiagnostic and histological examinations were not performed on this patient. These tests are essential for objective, evidence-based monitoring of adverse drug effects.

## Conclusions

In this case report, we discuss a patient with grade 3 peripheral neuropathy induced by oxaliplatin that developed rapidly after treatment switching. Supportive care was not effective because of the side effects of supportive care medicine and refusal of drugs due to side effects in this patient. Patients who are administered oxaliplatin-based chemotherapy for a long period of time should be monitored for the coasting phenomenon after the treatment is changed. There is no clinical evidence for the preventive effectiveness of the coasting phenomenon. Hence, investigation of predictors of the coasting phenomenon and adequate treatments are needed.
